# SCREENER, an educational game for teaching the Drug Discovery and Development process

**DOI:** 10.1590/1414-431X2021e11786

**Published:** 2021-12-03

**Authors:** F. Noël, G. Xexéo, E. Mangeli, A. Mothé, P. Marques, J. Kritz, F. Blanchard, H. Vermelho, B. de Paiva

**Affiliations:** 1 Laboratório de Farmacologia Bioquímica e Molecular, Instituto de Ciências Biomédicas, Universidade Federal do Rio de Janeiro, Rio de Janeiro, RJ, Brasil; 2 Programa de Pós-Graduação em Farmacologia e Química Medicinal, Universidade Federal do Rio de Janeiro, Rio de Janeiro, RJ, Brasil; 3 Instituto Nacional de Ciência e Tecnologia em Fármacos e Medicamentos, Rio de Janeiro, RJ, Brasil; 4 Laboratório de Ludologia, Engenharia e Simulação, Programa de Engenharia de Sistemas e Computação, COPPE, Universidade Federal do Rio de Janeiro, Rio de Janeiro, RJ, Brasil; 5 Departamento de Ciência da Computação, Instituto de Matemática, Universidade Federal do Rio de Janeiro, Rio de Janeiro, RJ, Brasil; 6 Instituto de Computação, Universidade Federal do Rio de Janeiro, Rio de Janeiro, RJ, Brasil; 7 Escola de Belas Artes, Universidade Federal do Rio de Janeiro, Rio de Janeiro, RJ, Brasil; 8 Escola Politécnica, Universidade Federal do Rio de Janeiro, Rio de Janeiro, RJ, Brasil

**Keywords:** Drug discovery, Education, Game, Pharmacology, Medicinal Chemistry, Drug development

## Abstract

Although the use of games as an educational strategy is an important current trend, there is practically no option available for training people on the Drug Discovery and Development (DDD) process. To fill this gap, we designed “SCREENER”, a science game that is intended to be educational, but also challenging and interesting enough to ensure player engagement. Our main target audience is students of postgraduate programs in pharmacology, medicinal chemistry, pharmacy, and medicine. This game could also be of interest to the pharmaceutical industry and regulatory and patent agencies for training new employees. We discuss the creation of SCREENER, a hybrid of board and card games, and present its components with some examples of cards and resources, as well as the dynamics of the game. SCREENER mimics the process of drug discovery and development from validating a target to registering the new drug with the regulatory agency, and can be played individually (self-learning) or with the help of a monitor who assists up to six players/teams. Briefly, 29 task cards categorized in four major areas (efficacy, safety, pharmacokinetics, and pharmaceutical development) must be purchased sequentially. Classic characteristics of games such as decision making and challenge have been incorporated. More in-depth information on the tasks and technical terms is available through QR codes. The vagaries of the DDD process are mimicked by the bonus/setback cards. The evaluation of our first test with students is presented and supports the usefulness of this new tool.

## Introduction

The process of Drug Discovery and Development (DDD) within a pharmaceutical industry, from validating a target to registering the new drug product with the regulatory agency, is particularly long and costly (1). This process is inherently multidisciplinary, and involves professionals from different fields, such as pharmacology, medicinal chemistry, toxicology, pharmaceutical technology, clinical investigation, and regulatory affairs.

Unfortunately, the lack of educational programs and qualified professionals with experience in all phases of the DDD process has been reported as one of the obstacles preventing progress in Brazilian capacity to provide innovative drugs, along with a lack of innovation culture (2). As a result, there is a need for educational initiatives, at least for graduate students somehow involved in DDD projects, to train them for a job market that is important worldwide and growing in Brazil. Indeed, the Brazilian government, through the focus of its funding agencies on translational research, as well as academia and several Brazilian companies have made considerable progress in the search for innovations in the pharmaceutical sector in the last fifteen years (2). More recently, independent initiatives have emerged, such as the accreditation of CQMED as an EMBRAPII unit for drug innovation (EMBRAPII is a social organization that supports technological research institutions fostering innovation in Brazilian industry).

It is well-known that active learning increases student performance in different areas (3). More specifically, the use of games as a strategy for educational purpose is an important current trend (4) and has been applied in different disciplines, including pharmacology (5). While serious games are becoming increasingly popular for teaching scientific concepts or themes (4,6), we identified only one commercially available board game on drug discovery and development: The PHARM GAME: Drug Discovery and Development (available at: https://squareup.com/market/thelearningkey/), but its high price is a major limitation at least for Brazilian graduate programs.

Faced with this panorama, we designed a game called “SCREENER” to fill this gap. Our intention was to create a science game that would be educational, but also challenging and interesting enough to ensure player engagement. We also decided to perform the first version in Portuguese to avoid any language barrier that could hamper the widespread use of this game, knowing that the English skills of undergraduate and graduate students in Brazil is often relatively poor (7). Our main target audience was students enrolled in postgraduate programs in pharmacology, medicinal chemistry, toxicology, pharmacy, and medicine as they can benefit from understanding that DDD is a team effort of individual scientists with highly specific expertise working on the same project. Our game can also be of interest to the pharmaceutical industry and regulatory and patent agencies for training new employees. Indeed, new employees of such companies and agencies often have a limited perspective of the complexity of the DDD process.

Here, we discuss the creation of SCREENER, a hybrid of board and card games, and present its components with some examples of the cards and resources as well as the dynamics of the game. Finally, we discuss a preliminary survey of students.

## Material and Methods

### Design and development process

SCREENER has been conceived as a collaborative project between people with experience on the theoretical aspects of the theme (8-10), experts in ludology with prior experience in game designing and production ([Bibr B11] [Bibr B12] 13), and people from the artistic design field. The idea matured after five years of using a board game in a discipline of the Postgraduate Program in Pharmacology and Medicinal Chemistry (PPGFQM) at the Federal University of Rio de Janeiro, Brazil. Our experience with MeduMAZE^TM^, a “*board game for training participants about the development and approval process for new medicines*” that was kindly provided by its creator (Dr. Alison Bowers), was very fruitful, but we felt that we should create our own game to better meet our needs. Basically, we wanted to reduce the need for a qualified monitor to explain all the stages of the process, increase the amount of information, especially about the discovery phase that our program is involved in, and use Portuguese to remove any language barrier, at least for the first version aimed to our main target audience.

### Dynamics and components

**Figure 1:**
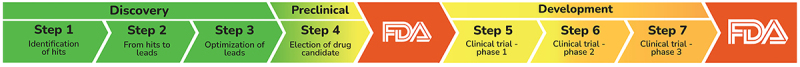
Map of the Drug Discovery and Development process. This map is used as a repository for the 29 task cards and two Federal Drug Administration (FDA) cards that are collected during the game. Translation of the original map in Portuguese.

**Figure 2:**
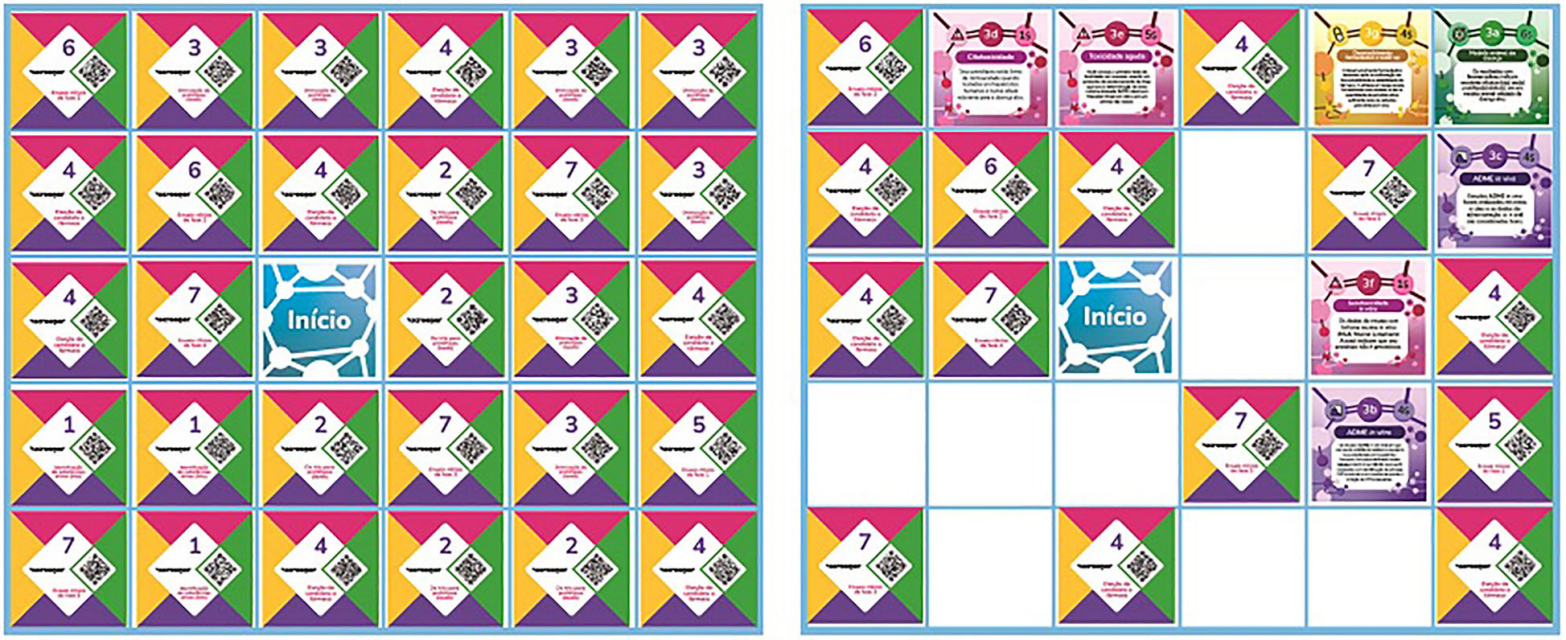
Grid (board) with the task cards in Portuguese. Left panel: representation of the board at the beginning of a game, with the initial card and the 29 task cards (face down). Right panel: representation of a board during a putative game, with some empty spaces, cards that have been turned over (face up), and cards in their original state (face down).

**Figure 3:**
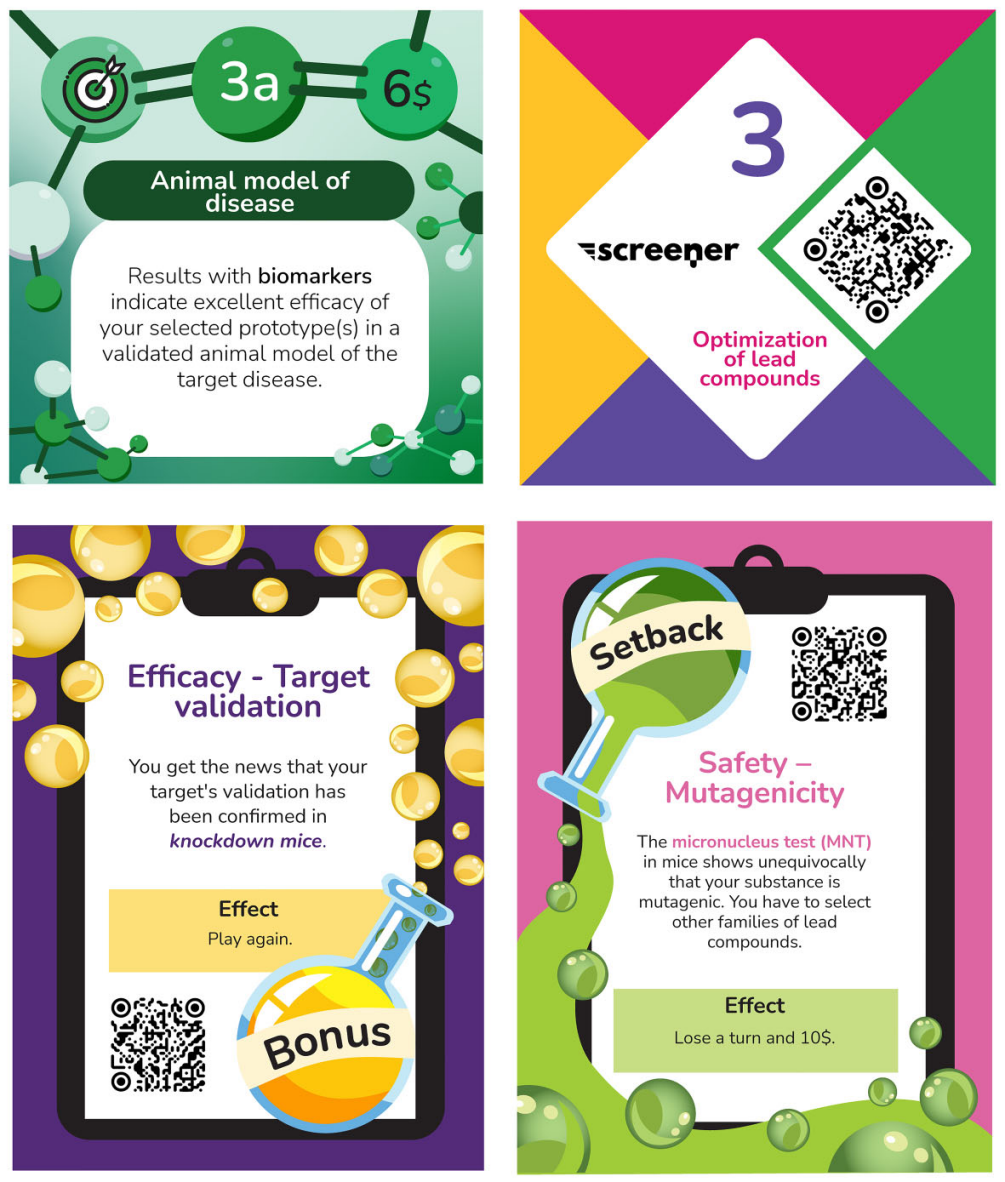
Examples of task cards and bonus/setback cards. Upper left panel: Front of a task card with logo and color indicating that the task is related to an efficacy test, number and letter identifying the stage and task, logo indicating the cost of the card, name of the task, and description of the result. Upper right panel: back of a task card showing the game logo, stage number, and QR code. Lower panels: front of a Bonus (left) and a Setback (right) card. Translation of the original cards in Portuguese.

The game was designed in such a way that it can be played individually (self-learning) or with the help of a monitor, assisting up to six players (or teams). Each player represents a company or team trying to register a new drug product. For this, it is necessary to fulfill all the tasks of each of the seven stages that are part of the DDD process, as detailed in the process map (Figure 1). The goal is to have a new drug product approved by the regulatory agency (Food and Drug Administration). Despite being competitors, all players collaborate to collect a single set of the 29 task cards that have to be found and purchased in the right order. A rules manual details all the steps and circumstances of the game. Briefly, the 29 task cards are placed face down on the grid (game board), as shown in Figure 2 (left panel). Players roll the dice and walk their playing piece up to the task card they need to pick up at that time, counting one step for each card or empty position on the grid. The right panel in Figure 2 shows a putative board during a game, with some empty positions (where cards have already been ‘bought' by a player), some cards turned over (face up), and some cards in their original state (face down). The tasks are categorized into four major areas (efficacy, safety, pharmacokinetics, and pharmaceutical development) identified by a color and a logo (Figure 3). All cards have a QR code that allows access to explanatory text in which some words may be highlighted to indicate that they belong to the 36 technical terms whose description can be accessed by touching the mobile phone screen or by looking them up in the glossary of the book.

At the beginning of the game, each player receives banknotes and a power card. The banknotes are necessary to ‘buy' task cards and to ‘pay' for possible setbacks that can occur when the player rolls the number 6 and must take a card that can be favorable (bonus card), unfavorable (setback card), or neutral (Figure 3). The four banknotes represent scientists with recognized achievements in drug discovery, namely: Sérgio Henrique Ferreira, Gertrude Belle Elion, Youyou Tu, and Paul Ehrlich. The six power cards have different moments of use and effects and relate to the following keywords: industrial espionage, patent law, outstanding scientist, contact network, investor partner, and marketing. The accompanying book (see discussion) describes real-life stories related to these power cards to illustrate, for example, the reality of the practice of industrial espionage: in 1997, Hsu Kai-Lo and Chester H. Ho, who were associated with a Taiwanese company, were arrested by the FBI and confessed that they had attempted to steal certain technology developed by the Bristol-Myers Squibb company to obtain commercial quantities of Taxol, the active ingredient in their drug product Paclitaxel^®^.

An important characteristic common to all games is that players must take actions to change the state of the game, and this action must be the result of a decision that enables or accelerates victory (14). In SCREENER, we incorporated three levels of decisions: 1) At the beginning of the game, each player chooses the kind of medicine he wants to develop; for example an immediate-release tablet containing a dopamine D3 receptor antagonist for the treatment of schizophrenia or an ointment containing an antiparasitic drug without a predefined molecular target for the treatment of cutaneous Leishmaniasis. These characteristics may eventually direct a player's response to or the outcome of a bonus/setback card; 2) during play, a player must decide each time he rolls the dice how best to obtain the task card of the time; and 3) the player must also decide when best to use his power card and challenge another player.

Another inherent characteristic of a game is that players can attack their opponents (15). In SCREENER, this occurs when a player draws a task card with a word printed in bold indicating that it is a technical term from the glossary. Any player can challenge the player who ‘buys' the card and force him to define the term. If the challenged player's explanations are deemed satisfactory based on the information available through the QR code, he receives a value; if not, he must ‘pay' that value.

### Playtesting

Parallel to the year-long development process, SCREENER was virtually tested using the “Tabletop Simulator” platform to comply with the social distancing regulations imposed by the COVID-19 pandemic. A final black-and-white draft version was then field-tested as part of a regular discipline offered to students in the Postgraduate program in Pharmacology and Medicinal Chemistry (PPGFQM-UFRJ), held in person in May 2021.

### Peer-review

All texts (book and rules manual) were reviewed by a professor of the PPGFQM. The book contains a short introduction on the DDD process and on the objectives of SCREENER as well as all the texts of the 29 task and 58 bonus/setback cards, including the information accessible through the QR codes. The glossary contains explanations for 36 technical terms (also available through the QR code). The contents of the six power cards are also described, along with real-life situations that inspired these situations. We also included a brief presentation of the four scientists chosen to illustrate the notes and the rationale for determining the cost of tasks.

### Production and availability

SCREENER will be available in both a free black and white version for printing at home (www.screener.com.br) and a color version produced by a professional print service. The color version will be distributed by the Brazilian Society of Pharmacology and Experimental Therapeutics (SBFTE) to the target audience, mainly postgraduate programs in pharmacology.

### Students' evaluation of the game

To assess the quality and usefulness of the game SCREENER for our purpose, we used the MEEGA+ questionnaire, developed especially for evaluating educational games (16-18). Students completed the online survey (in Portuguese) anonymously consisting of thirty-six close-ended unipolar items, with Likert-type 5-point answer options (“Strongly disagree”, “Disagree”, “Indifferent”, “Agree”, and “Strongly agree”).

## Results

Based on our first real-life experience, the estimated time for reaching product launch and completing the game is around 7-8 h, assuming six players and depending on the discussions that must be stimulated before accessing the QR codes. We used SCREENER as a tool for a regular postgraduate course that was divided as follows: an initial online meeting to introduce the players, explanations of the game and its rules, eventual formation of teams (6 to12 players), decision on the product each team will develop, and provision of a reference paper to be read before starting the game. In the next session, the game was played during three meetings of around 2-3 h. The fifth meeting was used for a formal Power-point presentation by the professor to review the sequence of events of a DDD project. The last meeting was reserved for short presentations by the six teams on topics they wanted to address.

As a preliminary student survey, the six participants in the first real-life test (PPGFQM, May 2021) completed the validated MEEGA+ questionnaire anonymously. Although we were aware that this first evaluation was very limited, preliminary, and qualitative, it indicated that the overall acceptance of the game was very good (large majority of “Agree” and “Strongly agree” answers). The evaluation of the dimensions social interaction, fun, focused attention, relevance, and perceived learning should be emphasized. On the other hand, some items received less positive evaluations: three items related to prior knowledge required, clarity of rules, and confidence that the game would be easy. A second group of items related to the relative lack of challenge and monotony and another to the feeling that personal effort was not so crucial.

## Discussion

It is argued that card and board games improve communication skills and promote active learning, which is why they are becoming increasingly popular in various fields, including medicine (19). On the other hand, such games for the DDD process are scarce. We did find game-based educational initiatives by three major pharmaceutical companies, but these were not intended for general use or were discontinued. Novartis created DRUG DISCOVERY AND DEVELOPMENT PROJECT SIMULATION for internal use as a tool for teaching new employees and, on rare occasions, for dissemination, such as during a pre-congress event of the 17th World Congress of Basic and Clinical Pharmacology held at Cape Town in July 2014, completed by one of the authors. With a general educational purpose, Boehringer Ingelheim created a Facebook game (SYRUP) and Eli Lilly an on-line board game (DESTINATION DISCOVERY), but both were discontinued. Based on their name, we found two other games that could be of interest for our purpose, but one of them is no longer working (DEVELOP A DRUG, developed by a science education center based at the Whitechapel Campus of Queen Mary University of London) and the other, BIG PHARMA, is a PC game developed by Twice Circled that deals with strategic business decisions, marketing, and malpractice in the pharmaceutical industry, rather than DDD (20). The only game commercially available that could attend our demand was THE PHARM GAME: DRUG DISCOVERY AND DEVELOPMENT, available at The Learning Key, Inc. and presented as a “*learning tool to teach novice and experienced employees about all aspects of pharmaceutical drug discovery and development*” (https://squareup.com/market/thelearningkey/). Unfortunately, its price (US$2,495) is an insurmountable limitation for its evaluation and potential use, at least for most Brazilian postgraduate programs.

Unlike most evaluations that use mainly qualitative methods for data analysis, we needed a more rigorous evaluation to somehow validate the quality and usefulness of SCREENER for the proposed purpose. For this reason, we used the MEEGA+ questionnaire, a systematic model originally developed by Petri et al. (16,18) to evaluate the perceived quality of educational games from the students' perspective. This model evaluates games in terms of quality factors and a set of nine dimensions.

We conclude that SCREENER is a very original educational board-cards game with online resources, that aims to teach the DDD process, including the sequence of stages and tasks to be performed, as well as the correct terms and related concepts of this multidisciplinary teamwork, preliminarily validated using a state-of-the-art methodology. It is expected that this tool will stimulate the creation of postgraduate disciplines in programs of pharmacology, pharmaceutical sciences, and medicinal chemistry.
